# Recombinant production of medium- to large-sized peptides in *Escherichia coli* using a cleavable self-aggregating tag

**DOI:** 10.1186/s12934-016-0534-3

**Published:** 2016-08-05

**Authors:** Qing Zhao, Wanghui Xu, Lei Xing, Zhanglin Lin

**Affiliations:** 1 Department of Chemical Engineering, Tsinghua University, One Tsinghua Garden Road, Beijing, 100084 China; 2 Novozymes, China Headquarters, 14 Xinxi Road, Shangdi Zone, Haidian District, Beijing, 100085 China; 3 China National Petroleum & Chemical Planning Institute, 16th Floor, 7 Block, Hepingli Zone, Beijing, 100013 China

**Keywords:** Therapeutic peptide production, Cleavable self-aggregating tag, Thioredoxin fusion tag, Authentic N-terminus

## Abstract

**Background:**

Peptides have recently become attractive for therapeutic applications. However, efficient production of medium- to large-sized peptides (30–100 amino acids [aa]) remains challenging both by recombinant and chemical synthesis. We previously reported the formation of active enzyme aggregates in *Escherichia coli* cells induced by the short β-structured peptide ELK16 (LELELKLKLELELKLK) and developed a streamlined protein expression and purification approach. In this approach, a cleavable self-aggregating tag (cSAT) consisting of an intein molecule and ELK16 was used to release the recombinant peptides with reasonable purity from active aggregates.

**Results:**

In this work, we extended the cSAT approach to a generalized expression and purification solution for a set of medium- to large-sized peptides with important therapeutic uses, including human glucagon-like peptide 1 (31 aa), B-type natriuretic peptide (32 aa), exendin 4 (39 aa), chemokine (C–C motif) ligand 5 (also known as RANTES, 66 aa), stromal cell-derived factor 1α (67 aa), insulin-like growth factor 1 (70 aa), and leptin (146 aa). After intein-mediated cleavage, the soluble peptides were released directly into the supernatant while insoluble peptides could be refolded and purified by reverse phase high-performance liquid chromatography. Additionally, an N-terminal thioredoxin tag was added upstream of the target peptides, which can be removed by enterokinase cleavage, generating native N-terminus for target peptides. Final yields of the peptides ranged from 0.1 to 1.8 μg/mg wet cell weight at laboratory scale.

**Conclusions:**

The approach described in this study provides a fast and efficient route to express and purify peptides that are difficult or expensive to produce by chemical synthesis or by ordinary recombinant methods. It is particularly well suited for large peptides, peptides likely to be degraded, and peptides that have toxic effects on the host. It can greatly reduce the cost and time of downstream processing, and thus may be useful for both industrial manufacture and laboratory applications.

**Electronic supplementary material:**

The online version of this article (doi:10.1186/s12934-016-0534-3) contains supplementary material, which is available to authorized users.

## Background

Development of facile and efficient techniques for the production of proteins and peptides has always been an important goal of biotechnology. Proteins and peptides play a crucial role in the biopharmaceutical industry as therapeutics and diagnostics in a variety of treatments, including endocrine disorders, cancer, and infectious diseases [1, 2]. In recent years, interest in using peptides as therapeutic agents has increased because of their high activity per unit mass, great chemical and biological diversity, and low toxicity [3, 4]. *Escherichia coli* (*E. coli*) is a preferred choice for producing recombinant proteins and peptides under 30 kDa that do not require complex post-translational modifications. This is owing to its fast growth rate, high product yield, and ease of culture. Given its ease of use, approximately 30 % of the recombinant products on the market are produced using *E. coli* [2, 5–7]. However, producing peptides shorter than 100 amino acids (aa) in *E. coli* is challenging because these peptides are susceptible to degradation [8–11]. In contrast, successful chemical synthesis and purification of peptides longer than 30 aa is difficult and highly sequence dependent [12]. Given the potential therapeutic value of peptides, an approach that enables efficient recombinant production of 30–100 aa peptides in *E. coli* could be immensely beneficial.

To overcome the difficulties associated with expressing foreign genes in *E. coli*, a diverse range of fusion tags has been developed that enhances stability and facilitates purification [13, 14]. One emerging class of fusion tags relevant to producing recombinant peptides results in the sequestration of fused peptides in inclusion bodies, which confer resistance to degradation, high expression rates, and simple recovery [15]. These tags include the very hydrophobic bacterial ketosteroid isomerase (KSI) [16], the autoprotease N^pro^ of classical swine fever virus [9, 17], and the elastin-like polypeptide (ELP) [18, 19]. However, the above fusion tags are typically large (over 120 aa). Moreover, separation of the target peptide from the fusion tags may require harsh chemical cleavage methods (as for the KSI scheme), tedious refolding (as for the N^pro^ scheme), or multiple phase transition cycling steps (as for the ELP scheme). Previously, we reported that C-terminal fusions of the short β-structured self-assembling peptide ELK16 (LELELKLKLELELKLK) can induce the formation of highly active enzyme aggregates [20]. Based on this finding, we designed a streamlined protein expression and purification approach. Briefly, target proteins were fused at the N-terminus of a cleavable self-aggregating tag (cSAT) composed of an *Mxe* GyrA intein and ELK16. The fusion proteins assembled as insoluble aggregates, which were isolated by centrifugation, where the self-cleavage activity of the intein was retained. After dithiothreitol (DTT)-induced intein cleavage, the target proteins were released into the soluble fraction, where they could be easily separated from the remaining insoluble protein. This single-step purification approach is capable of producing proteins with high yield and reasonable purity while reducing the cost and time required for purification [21].

In this study, we further extended the approach to successfully produce several therapeutically important peptides with lengths ranging from 30 to over 100 aa in *E. coli* cells, including the glucagon-like peptide 1 (GLP-1, 31 aa) [22], B-type natriuretic peptide (BNP, 32 aa) [23], exendin 4 (Ex-4, 39 aa) [24], chemokine (C–C motif) ligand 5 (CCL5, also known as RANTES, 66 aa) [25], stromal cell-derived factor 1α (SDF-1α, 67 aa) [26], insulin-like growth factor 1 (IGF-1, 70 aa) [27], and leptin (146 aa) [28]. All these peptides are of human origin except for Ex4, which is from *Heloderma suspectum*. Based on the expression and intein-mediated cleavage results, the above peptides were classified into three groups corresponding to different production schemes. After DTT induced intein-cleavage, the soluble peptides were released directly into the supernatant, as illustrated by GLP-1. For peptides that were insoluble after cleavage, another scheme involving further refolding steps was undertaken, by which CCL5, SDF-1α, IGF-1, and leptin were successfully purified. Additionally, to facilitate with the peptide expression in prokaryotic *E. coli* cells and eliminate the N-terminal methionine residue, we incorporated the thioredoxin (Trx) encoded by the *trx*A gene of *E. coli* followed by an enterokinase cleavage site as N-terminal fusion tag to the target peptides [29, 30]. With the Trx fusion tag, GLP-1, SDF-1α, and the previously unexpressed BNP and Ex4 were all produced in sufficient amounts and released into the soluble fraction. Then, peptides with native N-terminus were generated by removing the Trx tag by enterokinase cleavage, verified by matrix-assisted laser desorption ionization mass spectrometry (MALDI-TOF MS) analyses. Final yields of the peptides ranged from 0.1 to 1.8 μg/mg wet cell weight at laboratory scale. The approach described here may be of particular interest for the recombinant production of medium- to large-sized peptides that are prone to proteolysis, toxic to the host, or in other aspects challenging to express in *E. coli* cells.

## Results

### Constructions of fusion proteins

**Fig. 1:**
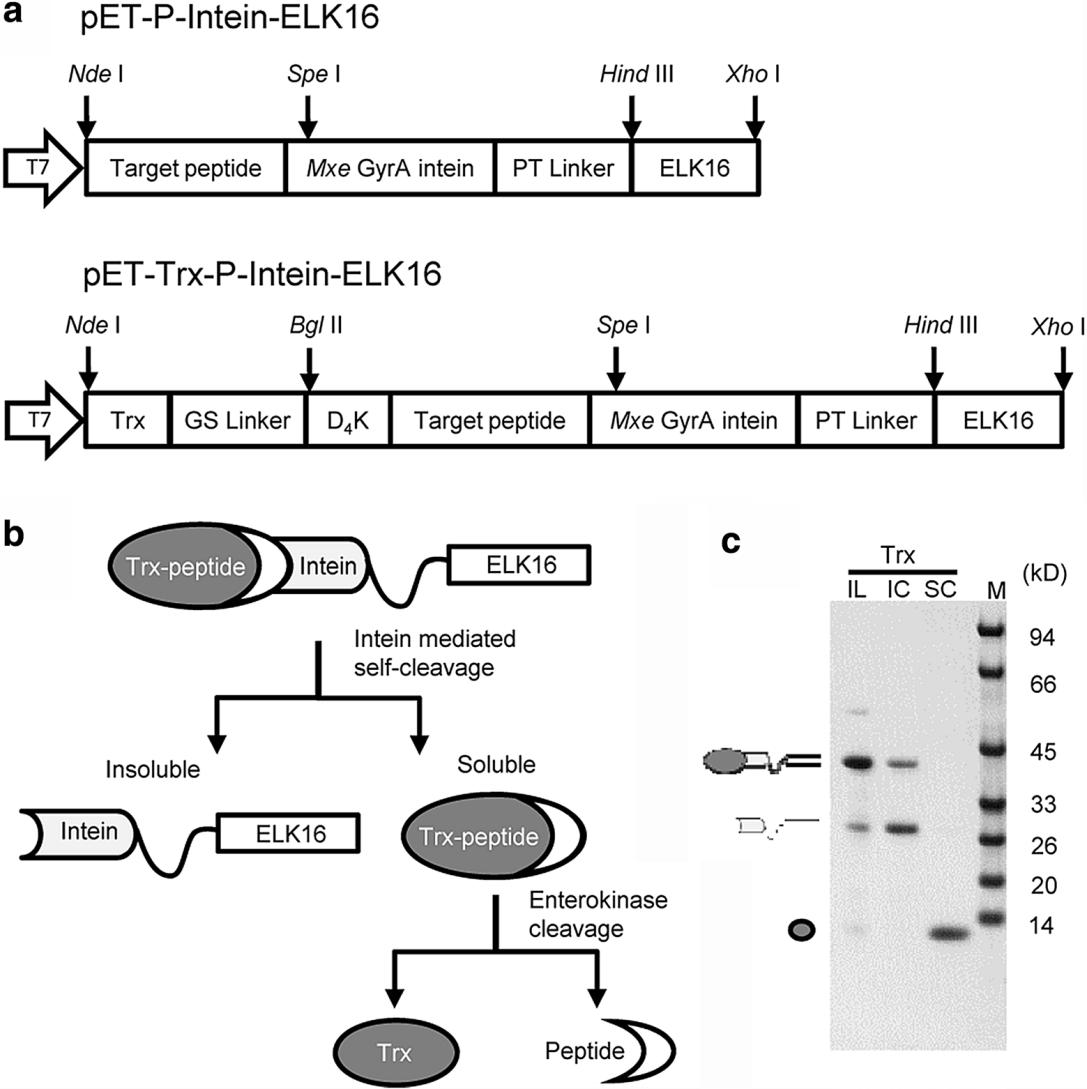
Construction of expression vectors. **a** The first expression vector pET-P-Intein-ELK16 was based on the previously constructed vector pET30a-LipA-I-ELK16. The second vector pET-Trx-P-Intein-ELK16 was derived from the first vector by inserting the thioredoxin (Trx) sequence followed by an enterokinase cleavage site Asp–Asp-Asp–Asp–Lys (D_4_K) upstream of the target peptide. **b** Schematic for producing recombinant peptides using vector pET-Trx-P-Intein-ELK16. **c** Expression and intein-mediated cleavage result of Trx. *Lane* IL, insoluble fraction of cell lysate after washing twice with buffer B1; *lanes* IC and SC, insoluble and soluble fraction of cleaved fusion protein

Two sets of vectors were constructed in this study, as shown in Fig. 1a. The first vector, pET-P-Intein-ELK16, was used to express the fusion protein peptide-intein-ELK16. It was based on a previously constructed vector pET30a-LipA-I-ELK16, where the intervening aa between the intein cleavage site and the target peptide C-terminus were removed [21]. The second vector, pET-Trx-P-Intein-ELK16, was modified from the first vector by inserting Trx (12.5 kD) encoded by the *trx*A gene of *E. coli* together with an enterokinase cleavage site Asp–Asp–Asp–Asp–Lys (D_4_K) upstream of the target peptide sequence. The scheme for producing recombinant peptides with the second vector is illustrated in Fig. 1b. Trx is one of the most commonly used fusion tags for improving protein expression and enhancing solubility in *E. coli* [31, 32]. Despite its high solubilization capacity, Trx can be directed into insoluble aggregates by ELK16 and then released into the soluble fraction after intein cleavage, as shown in Fig. 1c. pET-Trx-P-Intein-ELK16 was constructed to express the fusion protein Trx-peptide-intein-ELK16 to both improve the expression of peptides with difficult N-terminal sequences and increase the solubility of more hydrophobic peptides. Afterwards, the Trx tag was removed by enterokinase cleavage, generating the authentic N-terminus for the target peptide.

### Expression and intein-mediated cleavage of fusion proteins

**Fig. 2:**
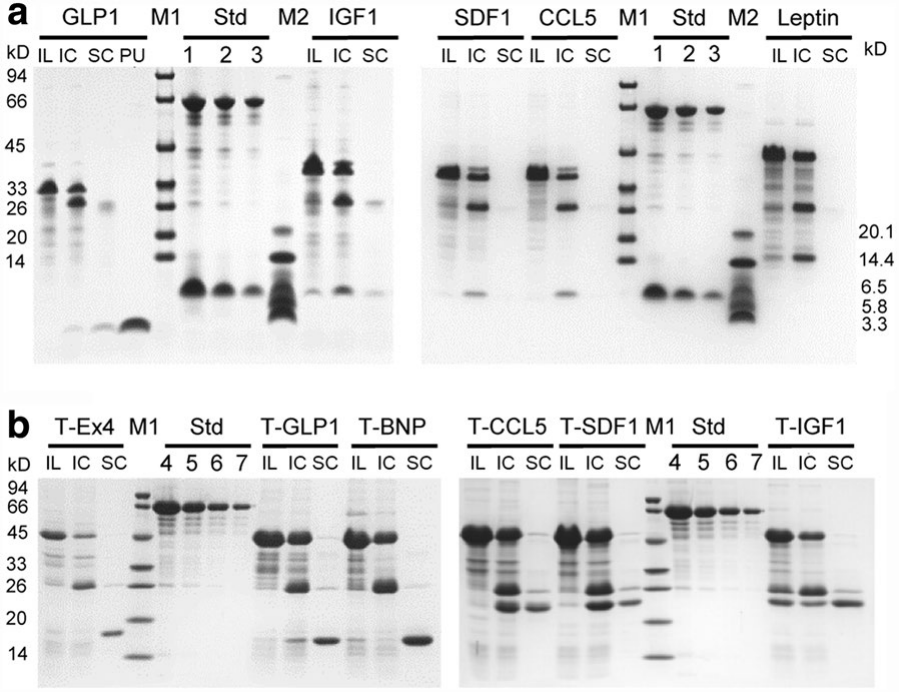
Expression and intein-mediated cleavage of fusion proteins. **a** Expression of five target peptides using the peptide-intein-ELK16 construct, designated with GLP1, IGF1, SDF1, CCL5, and Leptin. **b** Expression of six target peptides using the Trx-peptide-intein-ELK16 construct, designated with T-Ex4, T-GLP1, T-BNP, T-SDF1, T-CCL5, and T-IGF1. *Lane* IL, insoluble fraction of cell lysate after washing twice with buffer B1; *lanes* IC and SC, insoluble and soluble fraction of cleaved fusion protein; *lane* PU, final product of target peptide after RP-HPLC purification; *lanes* *1*, *2*, and *3*, quantification standards (Std) consisting of bovine serum albumin (BSA, 66.5 kDa) at 3, 1.5, and 0.75 µg/lane and aprotinin (6.5 kDa) at 1. 5, 0.75, and 0.3 µg/lane respectively; *lanes* 4, 5, 6, and 7, quantification standards (Std) consisting of bovine serum albumin (BSA, 66.5 kDa) at 6, 3, 1.5, and 0.75 µg/lane, respectively. The molecular masses of the protein standards M1 and M2 are listed by the *left and right side* separately

Seven model peptides with lengths between 31 and 147 aa were used in this work, and the sequences are listed in Additional file 1. For the scheme lacking the N-terminal Trx fusion, the target peptides GLP-1, IGF-1α, CCL5, SDF-1α, and leptin accumulated approximately 28.4–44.6 μg/mg wet cell weight as insoluble aggregates when fused to the self-cleavable intein-ELK16 tag (Fig. 2a; Table 1). The aggregates were then isolated by centrifugation and subjected to cleavage with 40 mM DTT at 4 °C for 24 h. GLP-1 was successfully released into the soluble fraction, and the apparent molecular mass was consistent with the theoretical value (3.5 kDa), indicating no degradation. The yield of purified GLP-1 was estimated to be 1.8 μg/mg wet cell weight, with 46.8 % recovery (Table 1). The purity was estimated to be 47 % with the residual fusion partner intein-ELK16 as the major impurity. Afterwards, GLP-1 was purified to homogeneity by reverse phase-high performance liquid chromatography (RP-HPLC), with the final product yield estimated to be 0.8 μg/mg wet cell weight, or 2.2 mg/L of OD600 2.0 LB culture (Scheme 1A in Table 2).

**Table 1:** Protein quantitation

Target peptide	MW (kD)	Aggregate yield^a^ (μg/mg wet cell pellet)	Peptide yield^b^ (μg/mg wet cell pellet)	Cleavage efficiency^c^ (%)	Percent recovery^d^ (%)
pET-P-Intein-ELK16					
GLP-1	3.5	31.1	1.8	60.9	46.8
IGF-1α^e^	7.8	35.4	5.2	70.6	62.0
CCL5^e^	7.8	35.5	5.2	70.9	61.3
SDF-1α^e^	8.0	28.4	3.5	53.3	50.7
Leptin^e^	16.2	44.6	10.5	60.3	60.3
pET-Trx-P-Intein-ELK16					
Trx-GLP-1	16.9	52.4	9.7	57.4	46.2
Trx-IGF-1α	21.2	34.0	9.2	67.8	59.4
Trx-CCL5	21.2	57.8	7.0	44.8	26.5
Trx-SDF-1α	21.2	56.5	5.0	35.3	19.3
Trx-BNP	17.0	49.9	13.4	67.1	66.6
Trx-Ex-4	17.7	11.4	3.4	75.3	73.1

^a^Yield of protein aggregate

^b^Yield of free target peptide after intein-mediated cleavage from LB culture with wet cell weight of 2.66 ± 0.99 mg/mL, estimated by densitometry analysis software Quantity One

^c^Cleavage efficiency as calculated by dividing the amount of cleaved protein aggregate over that of the total aggregate before cleavage

^d^Percent recovery in terms of mass as calculated by dividing the mass of the free peptide after cleavage over that could be theoretically obtained from the respective protein aggregate, assuming a complete cleavage and release

^e^The peptides were in the insoluble fraction after intein-mediated cleavage

**Table 2:** Summary of peptide production and purification schemes

Target peptide	Final yield of respective schemes (μg/mg wet cell pellet)^a^		
	Scheme 1A	Scheme 1B	Scheme 2
	Step 1: Expression of fusion protein Peptide-Intein-ELK16	Step 1: Expression of fusion protein Peptide-Intein-ELK16	Step 1: Expression of fusion protein Trx-Peptide-Intein-ELK16
	Step 2: Intein-mediated cleavage	Step 2: Intein-mediated cleavage	Step 2: Intein-mediated cleavage
	Step 3: RP-HPLC	Step 3: Refolding	Step 3: Enterokinase cleavage
		Step 4: RP-HPLC	Step 4: RP-HPLC
GLP-1	0.8 (20.8 %)^b^	–	1.1 (25.2 %)
BNP	No expression	–	1.8 (42.2 %)
Ex-4	No expression	–	0.3 (26.5 %)
IGF-1α	Insoluble	0.3 (3.6 %)	Unwanted enterokinase site
CCL5	Insoluble	0.1 (1.2 %)	Insoluble
SDF-1α	Insoluble	0.4 (5.8 %)	0.2 (2.0 %)
Leptin	Insoluble	0.6 (3.4 %)	Insoluble

^a^Yield of target peptide after fine purification by RP-HPLC from LB culture with wet cell weight of 2.66 ± 0.99 mg/mL, quantified by the Pierce® bicinchoninic acid (BCA) assay kit

^b^The final recovery after RP-HPLC purification listed in parentheses and calculated by dividing the product peptide yield by the theoretical value from aggregate yield in Table 1

However, for IGF-1, CCL5, SDF-1α, and leptin, most of the target peptide remained in the insoluble aggregates after cleavage, with peptide yields between 3.5 and 10.5 μg/mg wet cell weight and recoveries between 46.8 and 62.0 % (Table 1). These insoluble peptides required subsequent refolding steps to be separated from the fusion partner intein-ELK16, as described in the following section. For peptides BNP and Ex-4, no obvious expression was detected, probably because the N-terminal sequences of the two peptides were difficult to express in *E. coli* (data not shown).

For the scheme including the N-terminal Trx fusion (Fig. 1b), six target peptides, including GLP-1, the previously unexpressed BNP and Ex-4, and the insoluble IGF-1, CCL5, and SDF-1α (please see above), were now all successfully expressed. Upon intein-mediated cleavage, for GLP-1, BNP, Ex4, and IGF-1α, the Trx fusion peptides were released mostly in the soluble fraction, with yields between 3.4 and 13.4 μg/mg wet cell weight (Fig. 2b; Table 1). The recovery ranged from 46.2 to 73.1 %, which was comparable to those without the Trx tag. However, for CCL5 and SDF-1α, only a small percentage of the cleaved Trx fusion peptides distributed in the soluble fraction. The yield was 7.0 and 5.0 μg/mg wet cell weight while the recovery decreased to 26.5 and 19.3 %, respectively (Fig. 2b; Table 1). Leptin was still insoluble even with the N-terminal Trx tag and was not included in the following experiments (data not shown).

### Enterokinase cleavage of Trx-peptide and purification of target peptides

**Fig. 3:**
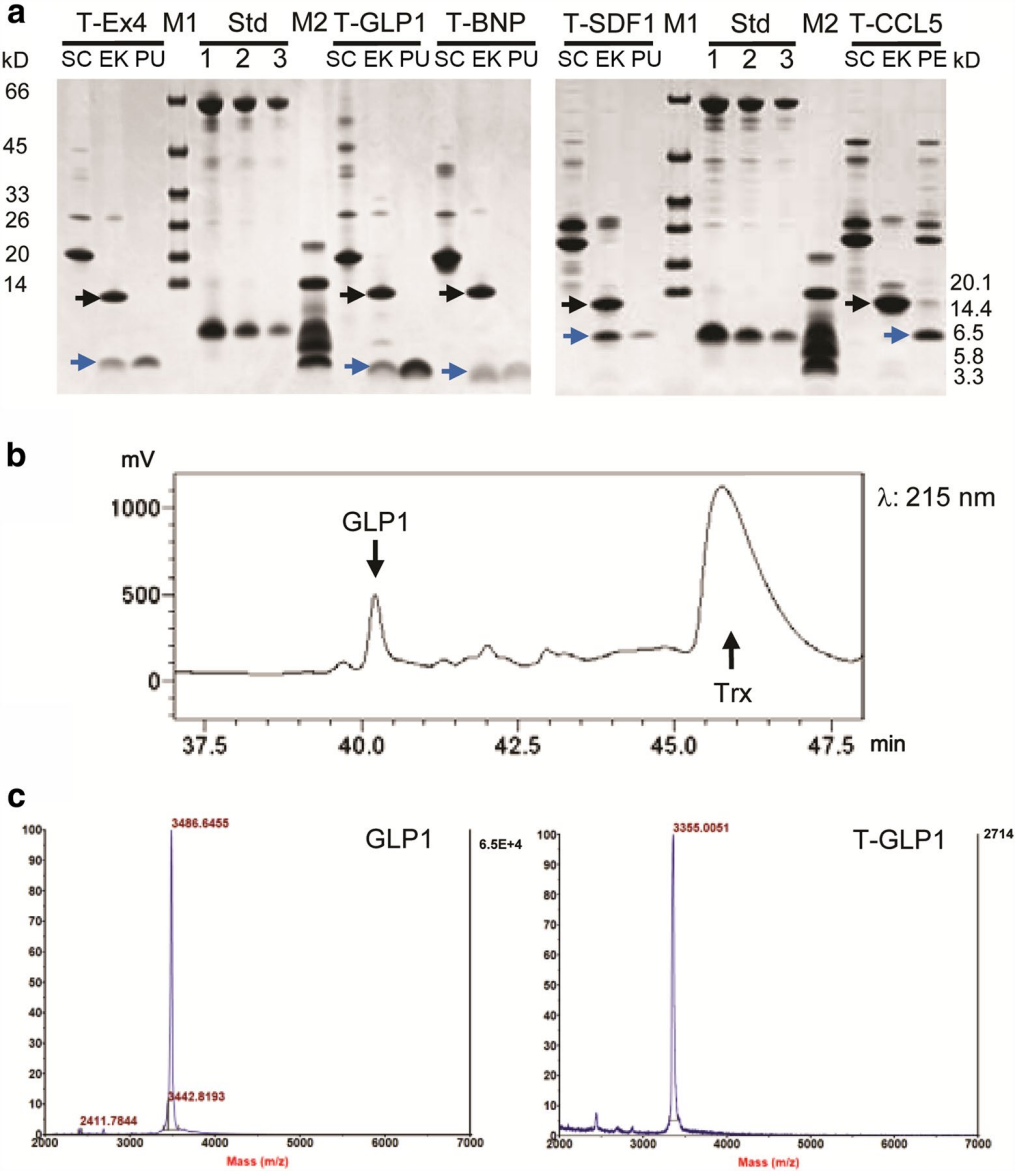
Production of target peptides with native N-terminus by removal of the Trx tag. **a** Enterokinase cleavage of Trx-peptide and purification results for different target peptides, designated with T-Ex4, T-GLP1, T-BNP, T-SDF1, and T-CCL5. *Black arrow* Trx; *blue arrow* target peptide. *Lanes* SC and EK, Trx-peptide before (soluble fraction of intein cleaved fusion protein) and after enterokinase cleavage; *lane* PE, precipitate after enterokinase cleavage of T-CCL5; *lane* PU, final product of target peptide after RP-HPLC purification; *lanes* 1, 2, and 3, quantification standards (Std) consisting of bovine serum albumin (BSA, 66.5 kDa) at 3, 1.5, and 0.75 µg/lane and aprotinin (6.5 kDa) at 1.5, 0.75, and 0.3 µg/lane, respectively. The molecular masses of the protein standards M1 and M2 are listed by the *left* and *right side* separately. **b** RP-HPLC separation of target peptide and Trx after enterokinase cleavage, illustrated by the chromatographic diagram of GLP-1. *X axis* retention time; *Y axis* peak height measured in mV. **c** MALDI-TOF analysis of GLP-1 produced using the vector pET-P-Intein-ELK16 (GLP1, *left*) or pET-Trx-P-Intein-ELK16 (T-GLP1, *right*)

For the target peptides that were expressed using the N-terminal Trx fusion, enterokinase cleavage was performed to remove the sequence upstream of the Trx tag. After reaction with 0.001 % (w/w) enterokinase at 23 °C for 16 h, almost 100 % of the Trx-peptide fusion proteins for GLP-1, BNP, Ex-4, CCL5, and SDF-1α were cleaved into two parts: the Trx tag and the target peptides (Fig. 3a). For IGF-1, however, cleavage occurred at a non-canonical enterokinase site, leading to a truncated peptide (data not shown). Except for CCL5, which accumulated in the insoluble fraction following enterokinase cleavage, the other four peptides remained soluble without the Trx tag (Fig. 3a). The soluble target peptides were then separated from the Trx tag by RP-HPLC, as exemplified by GLP-1 purification (Fig. 3b). Final yields of the peptides ranged from 0.2 to 1.8 μg/mg wet cell weight, corresponding to 0.5–4.9 mg/L OD600 2.0 LB culture (Scheme 2 in Table 2). The collected samples were then subjected to analyses by MALDI-TOF MS (see Additional file 2). For comparison, the MS value of GLP-1 expressed and purified by both methods was determined to be 3486.6/3355.0, consistent with the theoretical value of 3486.3/3355.1, which suggested correct processing by enterokinase (Fig. 3c).

### Refolding and purification of insoluble target peptides

**Fig. 4:**
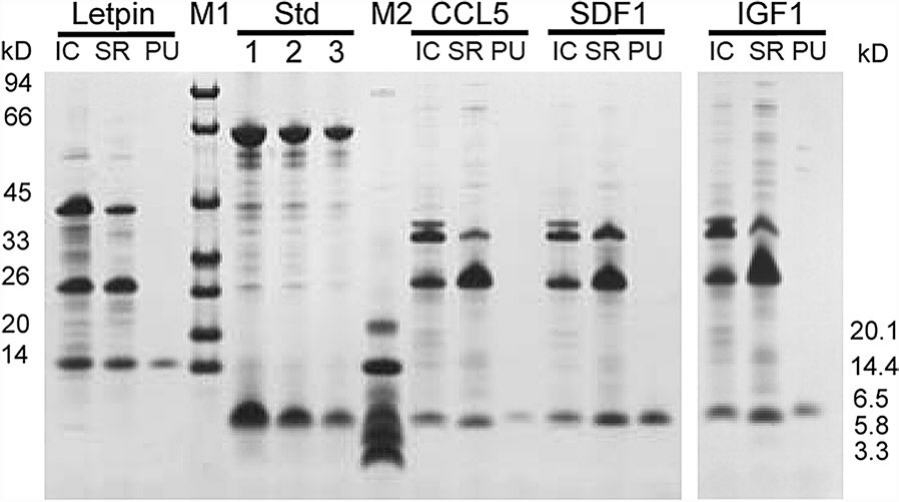
Refolding and purification of insoluble peptides. The refolding and purification results of different peptides, designated with Leptin, CCL5, SDF1, and IGF1. *Lane* IC, insoluble fraction after intein-mediated cleavage; *lane* SR, soluble fraction after refolding of the insoluble peptides; *lane* PU, final product of target peptide after RP-HPLC purification; *lanes* 1, 2, and 3, quantification standards (Std) consisting of bovine serum albumin (BSA, 66.5 kDa) at 3, 1.5, and 0.75 µg/lane and aprotinin (6.5 kDa) at 1.5, 0.75, and 0.3 µg/lane, respectively. The molecular masses of the protein standards M1 and M2 are listed by the *left* and *right side* separately

Refolding of the insoluble peptides IGF-1, CCL5, SDF-1α, and leptin was performed by unfolding the aggregates under chaotropic conditions (Buffer B1 with 6 M guanidine hydrochloride) followed by rapid 50-fold dilution in refolding buffer (Buffer BR). The insoluble debris of the diluted sample was removed by centrifugation and filtration, while the soluble fraction was then concentrated by ultrafiltration and subjected to SDS-PAGE (Fig. 4). All four target peptides were solubilized under this condition. At the same time, the fusion partner intein-ELK16 was also refolded and remained in the soluble fraction as the major impurity. The target peptides were further purified to homogeneity by RP-HPLC, with the final yield of 0.1 to 0.6 μg/mg wet cell weight, corresponding to 0.3–1.6 mg/L OD600 2.0 LB culture (Scheme 1B in Table 2).

## Discussion

In this work, we reported a generalized expression and purification solution for a set of medium- to large-sized peptides (between 30 and 150 aa) in *E. coli* cells based on a cleavable self-aggregating tag (cSAT) [20, 21]. The target peptides were produced and purified using three different schemes that accommodated a range of expression levels and hydrophobicity as summarized in Table 2. This approach features the efficient accumulation of a variety of target peptides into self-assembled aggregates and easy separation by intein-mediated cleavage, providing high expression rates and resistance to degradation.

The first scheme (Scheme 1A in Table 2) is especially advantageous for hydrophilic peptides that are prone to degradation when recombinantly expressed in *E. coli*, like GLP-1 in this study. GLP-1 is a gastrointestinal hormone with significant pharmaceutical importance in treating type 2 diabetes and is rapidly degraded by proteolytic enzymes when expressed in *E. coli* without modifications [33]. Previous *E. coli* expression and purification strategies for GLP-1 (using either a glutathione S-transferase (GST) or ubiquitin tag) require multiple processing and chromatography steps [33, 34]. In contrast, this strategy greatly simplified the expression and purification procedure, yielding a comparable recovery relative to that of the original fusion protein (the GST strategy, 31.6 %; the ubiquitin strategy, 5.4 %; our study, 20.8 %) [33, 34].

For many therapeutic peptides, the native N-terminus is essential and the initiator methionine must be removed. However, when recombinantly expressed in *E. coli*, the initiator methionine residue at the N-terminus of the target peptide usually remains owing to inefficient processing of the formyl-methionyl residue by endogenous deformylases and aminopeptidases [35]. In the second scheme (Scheme 2 in Table 2), an additional N-terminal fusion tag Trx was incorporated that could be removed by enterokinase cleavage. Trx is one of the most common fusion tags for improving protein expression in *E. coli* and has been reported to enhance the cytoplasmic solubility of proteins with disulfide bonds [36]. While BNP and Ex4 were not able to be expressed when using the first scheme, the fusion proteins Trx-BNP and Trx-Ex4 were efficiently expressed and then released as soluble fractions after intein-mediated cleavage. All four peptides produced by the second scheme, including GLP-1, BNP, Ex4, and SDF-1α, were obtained with correctly processed N-terminus, verified by MALDI-TOF MS analysis. Thus, the second scheme was very applicable to the target peptide that required a native sequence, or otherwise poorly expressed in *E. coli*.

However, some target peptides are prone to form aggregates and are unable to be released into the supernatant after intein-mediated cleavage. Cleaved peptides may remain insoluble owing to exposure of hydrophobic sequences, or the difficulty of forming correct double disulfide bonds in the cytoplasmic space in *E. coli*, like CCL5 (2 disulfide bonds), SDF-1α (3 disulfide bonds), and leptin (1 disulfide bond) in this work. In this case, the third scheme (Scheme 1B in Table 2) can be employed, which involves denaturation and refolding. As illustrated by SDF1-α, we discovered previously that the *E. coli* strain BL21(DE3) harboring the plasmid pET30a with SDF1-α sequence inserted in the MCS failed to produce the target band on SDS-PAGE (data not shown) and the chemical synthesis of this peptide also failed because of the long length. In this study, SDF1-α was produced as an aggregate and purified by refolding and RP-HPLC, with a final yield of 0.4 μg/mg. The recovery rate for this scheme was generally low compared with previous reports that involved large-scale fermentation and refolding [37–39]. The largest loss was due to the ultrafiltration step used in our refolding process, which gave only 20–50 % recovery. Even so, the third scheme provides a proof-of-concept means for the production of these peptides.

## Conclusions

Currently, downstream processing represents a major factor in terms of time and cost for the manufacturing of recombinant peptide or protein biopharmaceuticals. The approach we describe here provides a fast and efficient way to express and purify therapeutically important peptides that are difficult or expensive to produce by chemical synthesis or by ordinary recombinant methods. It may have the potential for both laboratory research work and industrial manufacture. The cSAT strategy described in this study may also be applicable to other industrial strains other than *E. coli.*

## Methods

### Materials

The DNA sequences encoding BNP, GLP-1, EX4, SDF1-α, CCL5, IGF-1, and leptin were optimized for expression in *E. coli* and synthesized by Genscript (Nanjing, China). Oligonucleotides for cloning were synthesized by Invitrogen (Shanghai, China). Restriction enzymes and DNA polymerases were purchased from New England Biolabs (Beverly, MA) or Takara (Dalian, China). The vector pET30a and strain *E. coli* BL21(DE3) were from Novagen (Madison, WI, USA). The kits for DNA purification, gel recovery, and plasmid mini-preparation were obtained from Tiangen (Beijing, China). Sequencing was performed by Invitrogen or by SinoGenoMax (Beijing, China). All other chemicals were of analytic grade.

### Construction of expression vectors

The target peptide sequence were inserted into the *Nde*I and *Spe*I sites of the previously constructed plasmid pET30a-LipA-I-ELK16, yielding the first expression vector pET-P-Intein-ELK16 [21]. For the second vector, the *trxA* gene (GenBank: AAA24534.1) encoding thioredoxin (Trx) was amplified from the *E. coli* BL21 (DE3) genome, digested with *Nde*I and *Spe*I restriction enzymes, and then ligated with the similarly digested pET30a-LipA-I-ELK16. The resultant plasmid pET-Trx-Intein-ELK16 contained a GS linker and an additional *Bgl*II site between the Trx and intein sequences. The target peptides were amplified with the introduction of an enterokinase site DDDDK at the 5′ end, digested with *Bgl*II and *Spe*I restriction enzymes and inserted into the digested pET-Trx-Intein-ELK16 with the same cohesive ends, yielding the second expression vector pET-Trx-P-Intein-ELK16. *E. coli* BL21 (DE3) was used throughout for cloning and protein expression.

### Expression and intein-mediated cleavage of fusion proteins

*Escherichia coli* BL21(DE3) cells harboring plasmid pET-P-Intein-ELK16 or pET-Trx-P-Intein-ELK16 were inoculated into Luria–Bertani (LB) medium supplemented with 50 mg/L kanamycin and incubated at 37 °C with shaking (250 rpm). Isopropyl β-D-1-thiogalactopyranoside was added to a final concentration of 0.2 mM to initiate protein expression when OD600 reached 0.4–0.6. The cultures were then continued for an additional 6 h at 30 °C (for the peptide GLP-1 and Ex4, expression was carried out at 37 °C for 6 h to achieve better expression), and then harvested by centrifugation at 6000×*g* for 10 min and pellets were stored at −70 °C for further assay and analysis.

Harvested cell pellets were re-suspended in buffer B1 (20 mM Tris–HCl, 500 mM NaCl, 1 mM EDTA, pH 8.5) to 10 OD culture/mL, followed by sonication (Ultrasonic crasher; Scientz JY92-IIN, Ningbo, China). The soluble fractions were isolated from the aggregates by centrifugation at 15,000×*g* for 15 min at 4 °C. The precipitates were washed twice with buffer B1, and re-suspended in the same volume of Buffer B3 (20 mM Tris–HCl, 500 mM NaCl, 1 mM EDTA, 40 mM dithiothreitol, pH 8.5). Intein-mediated cleavage reactions were performed by incubating the samples at 4 °C overnight. Then, the soluble and insoluble fractions were separated by centrifugation at 15,000×*g* for 15 min at 4 °C.

### Protein quantification

Protein samples were analyzed by denaturing polyacrylamide gel electrophoresis using 12 % SDS-PAGE gels or precast NuPAGE® precast 4–12 % Bis–Tris Gels from Invitrogen (Beijing, China), followed by staining with Coomassie Brilliant Blue G-250. The compositions and protein amounts of all samples were determined densitometrically with Quantity One software (Bio-Rad Laboratories, Hercules, CA, USA) using bovine serum albumin (BSA) and aprotinin as standards and adjusted according to the loading volume.

### Denaturation and refolding

The insoluble fraction after intein-mediated cleavage was re-suspended in Buffer BD (Buffer B1 containing 6 M guanidine hydrochloride) and incubated at 25 °C for 2 h. The insoluble debris was removed by centrifugation by centrifugation at 15,000×*g* for 15 min at 4 °C. Refolding was carried out by 1:50 dilution into Buffer BR (Buffer B1 containing 0.1 mM reduced glutathione, and 0.1 mM oxidized glutathione) with rapid stirring and then incubated at 4 °C for 24 h without further stirring. Insoluble debris containing unfolded proteins was removed by centrifugation at 15,000×*g* for 15 min at 4 °C and then filtered through a 0.25-μm membrane. The soluble fraction was desalted and concentrated by ultrafiltration using a Millipore Amicon^®^ Ultra-4 centrifugal filter device (3000 molecular weight cutoff).

### Reverse phase-high performance liquid chromatography (RP-HPLC)

The protein samples were applied to a BioBond™ C18 or C4 column (250 × 4.6 mm) (Dikma, Tianjing, China) connected to a Prominence LC-20A HPLC system (Shimadzu, Hong Kong, China), and eluted using a linear gradient from 5 to 80 % mobile phase B (Phase A: 0.12 % trifluoroacetic acid; Phase B: 80 % acetonitrile in water, 0.1 % trifluoroacetic acid) at a flow rate of 1 mL/min over 60 min. Target peaks were collected using an automatic fraction collector FRC-10A (Shimadzu), lyophilized and resuspended in water for MALDI-TOF MS analysis.

### Maldi-tof ms

MALDI-TOF MS was performed using the linear mode on an ABI 4800 plus MALDI-TOF/TOF MS Spectrometer by the Center of Biomedical Analysis, Tsinghua University.

## Additional files

10.1186/s12934-016-0534-3 Peptide sequences.
10.1186/s12934-016-0534-3 MALDI-TOF MS analyses of target peptides.

## Abbreviations

CCL5: chemokine (C–C motif) ligand 5
cSAT: cleavable self-aggregating tag
BNP: B-type natriuretic peptide
DTT: dithiothreitol
Ex-4: exendin 4
GLP-1: glucagon-like peptide 1
GST: glutathione S-transferase
IGF-1: insulin-like growth factor 1
SDF-1α: stromal cell-derived factor 1α
Trx: thioredoxin

## References

[1] Banga AK (2015). Therapeutic peptides and proteins: formulation, processing, and delivery systems.

[2] Walsh G (2014). Biopharmaceutical benchmarks 2014. Nat Biotechnol.

[3] Vlieghe P, Lisowski V, Martinez J, Khrestchatisky M (2010). Synthetic therapeutic peptides: science and market. Drug Discov Today.

[4] Fosgerau K, Hoffmann T (2015). Peptide therapeutics: current status and future directions. Drug Discov Today.

[5] Baeshen MN, Al-Hejin A, Bora RS, Ahmed M, Ramadan H, Saini KS, Baeshen NA, Redwan EM (2015). Production of biopharmaceuticals in *E. coli*: current scenario and future perspectives. J Microbiol Biotechnol.

[6] Kamionka M (2011). Engineering of therapeutic proteins production in *Escherichia coli*. Curr Pharm Biotechnol.

[7] Mora-Pale M, Sanchez-Rodriguez SP, Linhardt RJ, Dordick JS, Koffas MA (2014). Biochemical strategies for enhancing the in vivo production of natural products with pharmaceutical potential. Curr Opin Biotechnol.

[8] Albertsen L, Shaw AC, Norrild JC, Strømgaard K (2013). Recombinant production of peptide C-terminal α-amides using an engineered intein. Bioconjug Chem.

[9] Goda N, Matsuo N, Tenno T, Ishino S, Ishino Y, Fukuchi S, Ota M, Hiroaki H (2015). An optimized Npro-based method for the expression and purification of intrinsically disordered proteins for an NMR study. Intrinsically Disord Proteins.

[10] Hwang PM, Pan JS, Sykes BD (2014). Targeted expression, purification, and cleavage of fusion proteins from inclusion bodies in *Escherichia coli*. FEBS Lett.

[11] Wegmuller S, Schmid S (2014). Recombinant Peptide Production in Microbial Cells. Curr Org Chem.

[12] Bray BL (2003). Large-scale manufacture of peptide therapeutics by chemical synthesis. Nat Rev Drug Discov..

[13] Leder L, Freuler F, Forstner M, Mayr LM (2007). New methods for efficient protein production in drug discovery. Curr Opin Drug Discov Devel.

[14] Varga S, Pathare GR, Baka E, Boicu M, Kriszt B, Szekacs A, Zinzula L, Kukolya J, Nagy I (2015). Enhancing recombinant protein solubility with ubiquitin-like small archeal modifying protein fusion partners. J Microbiol Methods.

[15] Lin Z, Zhao Q, Xing L, Zhou B, Wang X (2015). Aggregating tags for column-free protein purification. Biotechnol J.

[16] Zhu X, Bi J, Yu J, Li X, Zhang Y, Zhangsun D, Luo S (2016). Recombinant Expression and Characterization of alpha-Conotoxin LvIA in *Escherichia coli*. Mar Drugs.

[17] Achmuller C, Kaar W, Ahrer K, Wechner P, Hahn R, Werther F, Schmidinger H, Cserjan-Puschmann M, Clementschitsch F, Striedner G (2007). N(pro) fusion technology to produce proteins with authentic N termini in *E. coli*. Nat Methods.

[18] Floss DM, Schallau K, Rose-John S, Conrad U, Scheller J (2010). Elastin-like polypeptides revolutionize recombinant protein expression and their biomedical application. Trends Biotechnol.

[19] Johnson T, Koria P (2016). Expression and purification of neurotrophin-elastin-like peptide fusion proteins for neural regeneration. BioDrugs.

[20] Wu W, Xing L, Zhou B, Lin Z (2011). Active protein aggregates induced by terminally attached self-assembling peptide ELK16 in *Escherichia coli*. Microb Cell Fact.

[21] Xing L, Wu W, Zhou B, Lin Z (2011). Streamlined protein expression and purification using cleavable self-aggregating tags. Microb Cell Fact.

[22] Lund A, Knop FK, Vilsbøll T (2014). Glucagon-like peptide-1 receptor agonists for the treatment of type 2 diabetes: differences and similarities. Eur J Intern Med.

[23] van Veldhuisen DJ, Linssen GC, Jaarsma T, van Gilst WH, Hoes AW, Tijssen JG, Paulus WJ, Voors AA, Hillege HL (2013). B-type natriuretic peptide and prognosis in heart failure patients with preserved and reduced ejection fraction. J Am Coll Cardiol.

[24] Kolterman OG (2003). Synthetic exendin-4 (exenatide) significantly reduces postprandial and fasting plasma glucose in subjects with type 2 diabetes. J Clin Endocrinol Metab.

[25] Appay V, Rowland-Jones SL (2001). RANTES: a versatile and controversial chemokine. Trends Immunol.

[26] Subramanian S, Liu C, Aviv A, Ho JE, Courchesne P, Muntendam P, Larson MG, Cheng S, Wang TJ, Mehta NN (2014). Stromal cell-derived factor 1 as a biomarker of heart failure and mortality risk. Arterioscler Thromb Vasc Biol.

[27] Collett-Solberg PF, Misra M (2008). The role of recombinant human insulin-like growth factor-I in treating children with short stature. J Clin Endocrinol Metab.

[28] Brennan AM, Mantzoros CS (2006). Drug Insight: the role of leptin in human physiology and pathophysiology–emerging clinical applications. Nat Clin Pract Endocrinol Metab.

[29] Wood DW (2014). New trends and affinity tag designs for recombinant protein purification. Curr Opin Struct Biol.

[30] Esposito D, Chatterjee DK (2006). Enhancement of soluble protein expression through the use of fusion tags. Curr Opin Biotechnol.

[31] Khalili M, Soleyman MR, Baazm M, Beyer C (2015). High-level expression and purification of soluble bioactive recombinant human heparin-binding epidermal growth factor in *Escherichia coli*. Cell Biol Int.

[32] Nguyen MT, Koo B-K, Vu TTT, Song J-A, Chong S-H, Jeong B, Ryu H-B, Moh S-H, Choe H (2014). Prokaryotic soluble overexpression and purification of bioactive human growth hormone by fusion to thioredoxin, maltose binding protein, and protein disulfide isomerase. PLoS ONE.

[33] Zhang ZZ, Yang SS, Dou H, Mao JF, Li KS (2004). Expression, purification, and C-terminal amidation of recombinant human glucagon-like peptide-1. Protein Expr Purif.

[34] Kim SG, Shin SY, Park YC, Shin CS, Seo JH (2011). Production and solid-phase refolding of human glucagon-like peptide-1 using recombinant *Escherichia coli*. Protein Expr Purif.

[35] Giglione C, Fieulaine S, Meinnel T (2015). N-terminal protein modifications: bringing back into play the ribosome. Biochimie.

[36] Kong B, Guo GL (2014). Soluble expression of disulfide bond containing proteins FGF15 and FGF19 in the cytoplasm of *Escherichia coli*. PLoS ONE.

[37] Veldkamp CT, Peterson FC, Hayes PL, Mattmiller JE, Haugner JC, de la Cruz N, Volkman BF (2007). On-column refolding of recombinant chemokines for NMR studies and biological assays. Protein Expr Purif.

[38] Jeong KJ, Lee SY (1999). High-level production of human leptin by fed-batch cultivation of recombinant *Escherichia coli* and its purification. Appl Environ Microbiol.

[39] Kim SO, Lee YI (1996). High-level expression and simple purification of recombinant human insulin-like growth factor I. J Biotechnol.

