# Supplementary material for: Recombinant production of medium- to large-sized peptides in Escherichia coli using a cleavable self-aggregating tag
Source: Microb Cell Fact. 2016 Aug 5;15:136. doi: 10.1186/s12934-016-0534-3 (PMC4975908; doi:10.1186/s12934-016-0534-3)
Supplement: Supplementary file 1 — 10.1186/s12934-016-0534-3 Peptide sequences. [file 12934_2016_534_MOESM1_ESM.pdf]

Additional file 1. Peptide sequences.

Peptide	Residue number (aa)	Sequence
**GLP-1**	31	HAEGTFTSDVSSYLEGQAAKEFIAWLVKGRG
**BNP**	32	SPKMVQGSGCFGRKMDRISSSSGLGCKVLRRH
**Ex-4**	39	HGEGTFTSDLSKQMEEEAVRLFIEWLKNGGPSSGAPPPS
**CCL5**	66	YSSDTTPCCFAYIARPLPRAHIKEYFYTSGKCSNPAVVFVTRKNRQVCANPEKKWVREYINSLEMS
**SDF-1α**	67	KPVSLSYRCPCRFFESHVARANVKHLKILNTPNCALQIVARLKNNNRQVCIDPKLKWIQEYLEKALN
**IGF-1**	70	GPETLCGAELVDALQFVCGDRGFYFNKPTGYGSSSRRAPQTGIVDECCFRSCDLRRLEMYCAPLKPAKSA
**leptin**	146	VPIQKVQDDTKTLIKTIVTRINDISHTQSVSSKQKVTGLDFIPGLHPILTLSKMDQTLAVYQQILTSMPSRNVIQ ISNDLENLRDLLHVLAFSKSCHLPWASGLETLDSLGGVLEASGYSTEVVALSRLQGSLQDMLWQLDLSPGC
