# Supplementary figures and images for: Recombinant production of medium- to large-sized peptides in Escherichia coli using a cleavable self-aggregating tag
Source: Microb Cell Fact. 2016 Aug 5;15:136. doi: 10.1186/s12934-016-0534-3 (PMC4975908; doi:10.1186/s12934-016-0534-3)

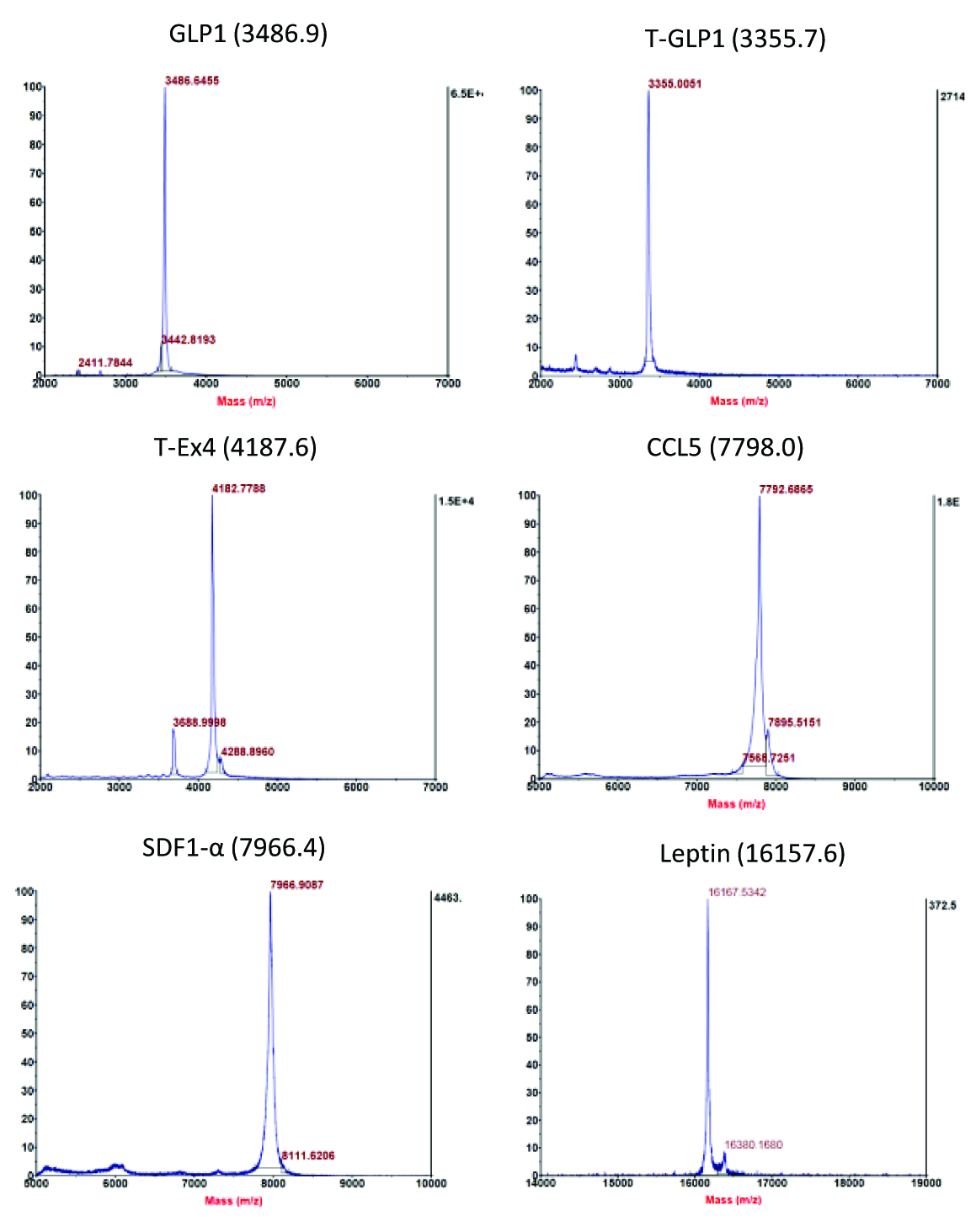

Supplement: Supplementary file 2 — 10.1186/s12934-016-0534-3 MALDI-TOF MS analyses of target peptides. [file 12934_2016_534_MOESM2_ESM.tif]
